# Performance-Guided Design of Permeable Asphalt Concrete with Modified Asphalt Binder Using Crumb Rubber and SBS Modifier for Sponge Cities

**DOI:** 10.3390/ma14051266

**Published:** 2021-03-07

**Authors:** Wentong Huang, Xiao Liu, Shaowei Zhang, Yu Zheng, Qile Ding, Bin Tong

**Affiliations:** 1 School of Environment and Civil Engineering, Dongguan University of Technology, Dongguan 523808, China; huangwt@dgut.edu.cn (W.H.); zhengy@dgut.edu.cn (Y.Z.); dingql@dgut.edu.cn (Q.D.); tongb@dgut.edu.cn (B.T.); 2 School of Architecture, South China University of Technology, Guangzhou 510641, China; 3 Architectural Design & Research Institute Co., Ltd., South China University of Technology, Guangzhou 510641, China; 4 State Key Laboratory of Subtropical Building Science, South China University of Technology, Guangzhou 510641, China; ctzsw@mail.scut.edu.cn

**Keywords:** crumb rubber, permeable asphalt concrete, resilient modulus, permeability coefficient, sponge city

## Abstract

The construction of sponge city is a major green innovation to implement the concept of sustainable development. In this study, the road performance of permeable asphalt concrete (PAC), which displays pronounced water permeability and noise reduction that are favorable for sponge cities, has been improved with a two-fold modification using styrene–butadiene–styrene (SBS) and crumb rubber (CR). Four percent SBS and three different ratios (10%, 15%, and 20%) of CR have been used to modify the virgin asphalt binder. The Marshall design has been followed to produce PAC samples. To evaluate the asphalt binder performance, multiple-stress creep-recovery (MSCR) test, linear amplitude sweep (LAS) test, and engineering property test programs including softening point test, penetration test, and rotational viscosity test have been conducted. Freeze–thaw splitting test, Hamburg wheel-tracking test, resilient modulus test, and permeability coefficient test have been performed to evaluate the asphalt mixture performance. The test results show that the addition of SBS and CR reduces the permeability coefficient, but significantly improves the high temperature performance, fatigue performance, and rutting resistance as well as the resilient modulus. However, the optimum rubber content should not exceed 15%. Meanwhile, after adding CR and SBS modifier, the indirect tensile strength (ITS) and tensile strength ratio (TSR) increase. It indicates that the moisture stability and crack resistance have been improved by the composite modification effect.

## 1. Introduction

**Figure 1:**
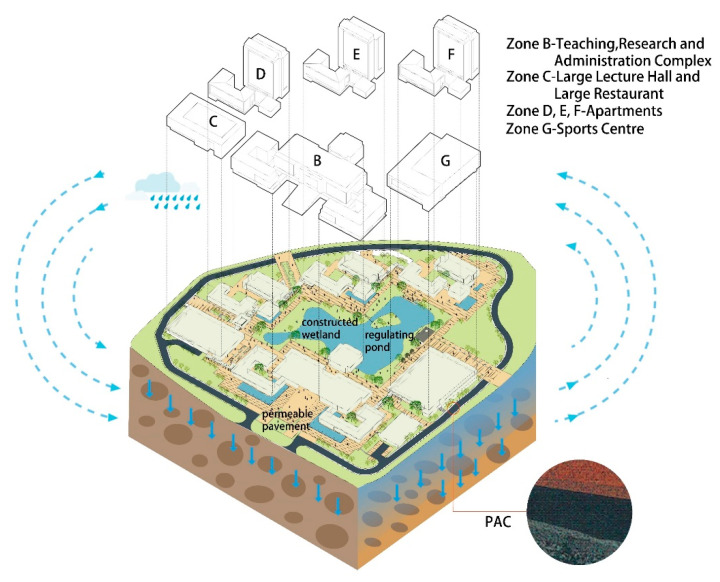
China Capital Market Institute (CCMI)—a good example of sponge city construction in Shenzhen.

A “sponge city” refers to cities with good resilience (like a sponge) in adapting to environmental changes and responding to natural disasters. That is, a sponge city functions well in natural retention, permeation, and purification [1]. China has been vigorously promoting contemporary sponge city construction. In this context, the infrastructure should actively introduce the low-impact development (LID) model that respects and follows nature for the construction of a sponge city. This means to make full use of permeable areas such as green spaces, roads, and water systems, etc., to absorb, retain and slowly release rainwater, reduce campus rainwater runoff volume and peak flow, slow down rainwater runoff speed, extend the duration of rainwater runoff generation, replenish groundwater, and purify rainwater pollutants [2]. As shown in Figure 1, through systematic “sponge” design, the China Capital Market Institute (CCMI) has realized multiple sponge city facilities including permeable pavements, integrated rainwater permeation and drainage systems, green roofs, rainwater wetlands, regulating pools (landscape lakes), reservoirs, rainwater gardens, etc., presenting a good example of sponge city construction in Shenzhen [3]. Especially, permeable asphalt concrete (PAC) is a new type of pavement with ecological and environmental benefits and a promising prospect in sponge city application. With a macroporous design, it can effectively reduce road surface runoff, and facilitate the retention and re-utilization of rainwater for the replenishment of groundwater. According to site monitoring and analysis of PAC conducted by Xu et al. in national demonstration zones of low impact development and comprehensive utilization of rainwater in Shenzhen Guangming New District, PAC can reduce total runoff and peak flow by 76.70% and 74.92% on average, respectively, when compared with conventional impermeable asphalt roads [4].

There have been many studies [5,6,7,8,9,10] on PAC due to its numerous benefits to road safety, driving comfort, and environmental value. Compared to conventional asphalt pavements such as dense asphalt mixture and stone matrix asphalt (SMA), etc., PAC is typically an open-graded porous mixture with a high air void ratio which is generally around 18–25% [11]. Notably, its specific structure design highly improves road safety because of the large existing interconnected porosity; during rainfall, the water can successfully flow out of the pavement system to avoid accumulating on the pavement surface, which can bring potential risks to driving safety [10]. Along with that, PAC has promising performance on improving skid resistance, and reducing hydroplaning and splashing, which can benefit road safety. Based on a report from Texas, with the PAC experimental section, there was a 51% reduction in car accidents which occurred in wet weather [12]. Similarly, researchers from Japan have found that using PAC can result in a significant drop in car accidents (i.e., 80%) during wet conditions [13]. From the perspective of road safety, these can well explain the popularity of PAC in developed countries.

There are studies [14,15,16] which have pointed out that tire–pavement noise is one of the major sufferings for travelers and passengers because of the voluble compressed air under the tires in the driving. As reported by Kandhal [17], on one hand, PAC can effectively reduce the generation of pumping noise. On the other hand, it can benefit the energy conversion from an acoustic manner to an internal manner; therefore, the “painful” feeling caused by the tire–pavement noise can be alleviated in a large context. Many studies have used PAC to develop low noise pavement and achieved favorable experimental results [18,19,20,21]. Anderson et al. [19] have mentioned that compared to the rubberized asphalt pavement, the performance of reducing the tire–pavement noise of PAC is more remarkable, and the average reduction in noise level is 4 dB. Beyond that, PAC has significant environmental value, which is deeply beneficial to our ecological society. Based on Chen et al. [22], PAC can alleviate the urban heat island (UHI) effect in megacities under extreme weather conditions, because the sponge structure can introduce thermal transmission between the top and bottom layers. 

Although PAC possesses the aforementioned advantages, a common conception from the researchers, industries and public officials is that PAC is easily affected by environmental conditions such as water and temperature. Compared to the dense-graded asphalt used daily, PAC requires a high viscosity on the asphalt grade; meanwhile, the large ratio of porosity also sacrifices the inferior strength of the pavement structure. Therefore, to some extent, PAC is restricted for practical uses with its high requirements in production. To address the issues of engineering properties among low water stability and the short service life of PAC, one possible solution is to use the modified asphalt binder to produce PAC to meet practical needs. Prominent researchers in asphalt modification have led systematic studies on crumb rubber/styrene–butadiene–styrene (CR/SBS) composite modified asphalt with continued efforts, which is a strong base for improving PAC’s engineering properties [23,24,25,26,27]. Based on Jin et al., different types of SBS such as linear SBS and radial SBS, different contents of SBS modifier, and sulfur content have impacts on the properties of the asphalt binder and asphalt mixture. Regarding the improvement of low temperature performance, the results show that under their experimental design, the optimum contents of SBS on asphalt binder and asphalt mixture are 6% and 4.5%, respectively [23,24]. Yu et al. have conducted a thorough analysis on wet-process rubberized asphalt binder with warm mix asphalt (WMA) additives and have found that it is effective in alleviating the hardening and cracking of the virgin asphalt binder, exhibiting a more durable service life for the asphalt mixture [25,26,27]. 

Indeed, a study from Japan has proposed the use of polymer modified asphalt (PMA) to improve the resistance of PAC’s rutting deformation; the presented experimental results demonstrated that it can successfully extend PAC’s service life [28]. Moreover, Murayama et al. [29] have evaluated the fatigue life of PAC using SBS modified asphalt binder. Specifically, four-point beam fatigue tests have been performed. A statistical model used to build the relationship between fatigue life and dissipated energy has been derived. The experimental results conclude that the high fatigue life of PAC can be a possible replacement, especially in rainy areas. Inspired by the abovementioned studies and those conducted by other researchers [30,31], which have investigated the superior cracking of rubberized asphalt binder, the authors have developed a new type of PAC which uses SBS and CR to modify the virgin asphalt binder. 

## 2. Research Objective and Scope

**Figure 2:**
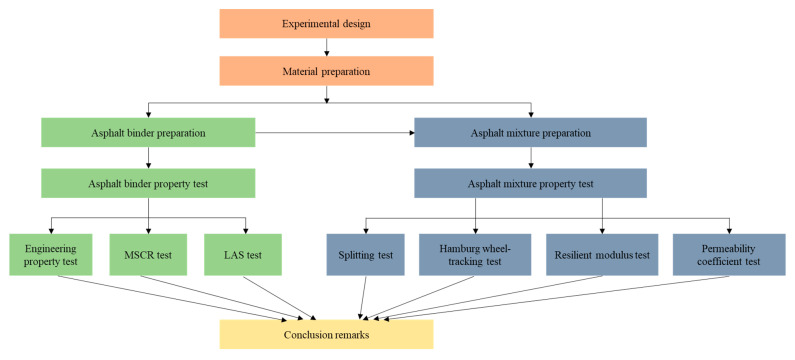
Overview of this study (Multiple Stress Creep Recovery (MSCR) and Linear Amplitude Sweep (LAS)).

In this study, to address the issues of short service life, low moisture stability, and poor crack resistance of conventional PAC, the research objectives were to: (i) comprehensively characterize and compare the rheological properties of the modified asphalt binder using SBS and CR modifiers; and (ii) fully investigate the mechanical performance of PAC using the modified asphalt binder. On the one hand, the modified asphalt binder leverages better rheological performance over a wider range of temperatures of the CR modifier. On the other hand, the modified asphalt binder can have improved fatigue and thermal cracking resistance by using the SBS modifier. In practice, the improved PAC can have better road performance and a longer service life with improved moisture stability and crack resistance. Regarding the modified asphalt binder, the engineering property tests (penetration, softening point, and rotational viscosity test), multiple-stress creep-recovery (MSCR) test, and linear amplitude sweep (LAS) test have been performed. To investigate and compare the mechanical properties of the improved PAC in a thorough manner, a freeze–thaw splitting test, hamburger wheel-tracking test, resilient modulus test, and permeability coefficient test have been conducted. Figure 2 shows the workflow of this study. 

## 3. Materials and Experimental Design 

### 3.1. Preparation of Sponge City (SC) Asphalt Binder

#### 3.1.1. Virgin Asphalt Binder

In this study, asphalt binder supplied by a local manufacturer, which followed a penetration grade of 60/70, was used. The test values among different properties including penetration, softening point, ductility, and density are shown below in Table 1. All the test approaches have followed and satisfied the requirements of Technical Specifications for the Construction of Highway Asphalt Pavement [32].

#### 3.1.2. The Modified Asphalt Binder

As mentioned above, the modified asphalt binder attempts a composite modification manner with the CR and SBS modifier. To generate the proposed asphalt binder, 40-mesh CR collected from a local supplier has been used, and its gradation is outlined in Table 2. Specifically, the very first step is to produce the rubberized asphalt binder. Based on Yu et al. [33], the wet mixing process is preferred because it involves reactions of the absorptions of aromatic oils from asphalt binder into the polymer chains that can cause the structural changes of the asphalt binder. In detail, the virgin asphalt binder needs to preheat to 180 °C and then add the properly weighted SBS modifier (4% content). After that, the industrial sulfur (stabilizer) is added for shear mixing (6000 r/min) before swelling for 1.5 h. The main reason for adding sulfur into the modified asphalt binder is that it can be dispersed in asphalt and replaces a significant ratio of asphalt binder in the application. In other words, drawbacks of polymer modified asphalt binder such as phase separation, low resistance to heat, oxidation, and ultraviolet can be alleviated. To finish the whole production process, adding the CR modifier before shear mixing for another 1.5 h is required. Three dosages with the CR modifier content being 10%, 15% and 20% have been adopted. 

### 3.2. Preparation of Porous Asphalt Concrete

**Figure 3:**
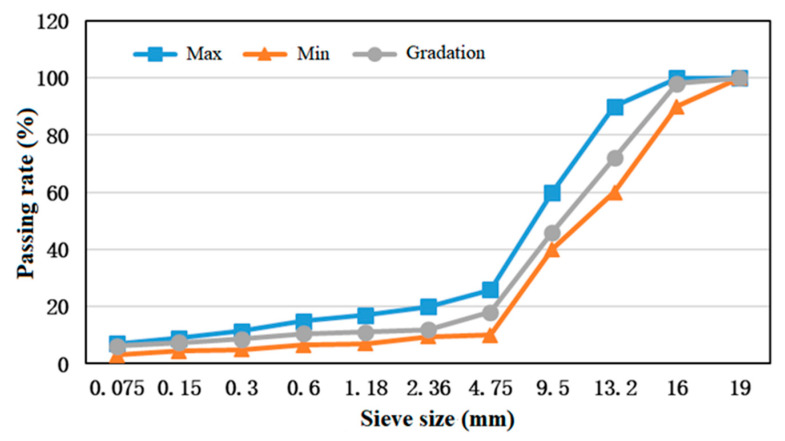
Gradation of porous asphalt concrete.

The mixture has been made with basalt of four different proportions, i.e., 0–3 mm, 3–5 mm, 5–10 mm, and 10–15 mm, and by adjusting the aggregate of each proportion with reference to the targeted mix of PAC-16, the final gradation curve is shown below in Figure 3.

The PAC specimens were produced based on the Marshall design following the Chinese protocol [32]. Asphalt content has been designated as 4.5%, 5%, 5.5% and 6%, respectively, for determining the optimum content. At the same time, 4% SBS, 4% SBS + 10% CR, 4% SBS + 15% CR, and 4% SBS + 20% CR have been incorporated as test groups for comparison. 

**Figure 4:**
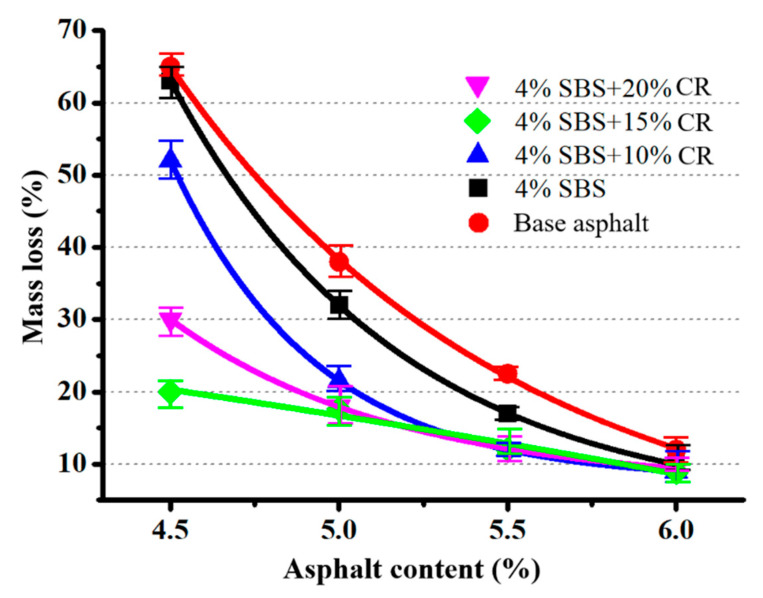
Diagram of the relationship between Cantabro mass loss and asphalt content.

Based on the experimental results, the relationship between mass loss and asphalt content can be found in Figure 4. It is easy to observe that the mass loss of the prepared PAC sample has reduced significantly when the asphalt content is in the low ratio domain. However, with the increase in asphalt content, the modification effect on mass loss is reduced. When asphalt content reaches 6%, no significant difference has been investigated in mass loss between the control group and the test groups. The reason could be that the addition of the modifier can improve the mechanical property of asphalt when its content is low. Moreover, the increased content of the virgin asphalt binder can introduce the improved viscosity between the asphalt binder and the aggregate. In other words, the modifier’s impact is limited when the asphalt content is high, leading to similar mass loss between the control group and the test groups. 

**Figure 5:**
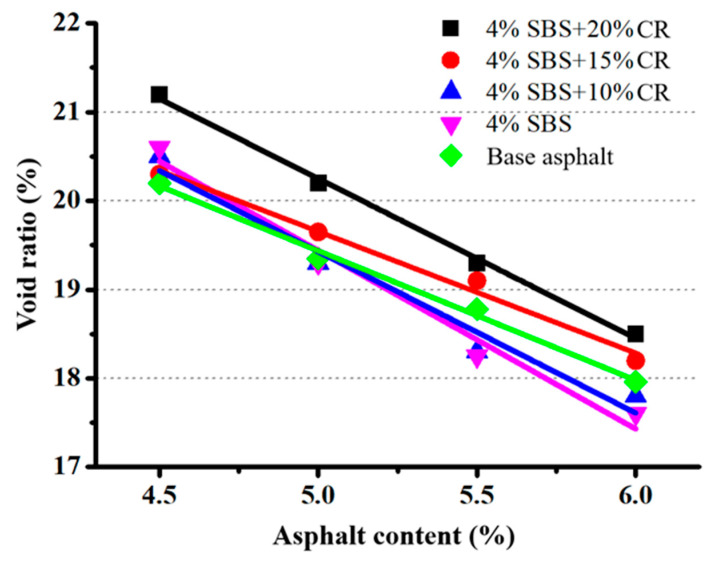
Relationship between void ratio and asphalt content.

Figure 5 presents the relationship between the void ratio of the PAC sample and the asphalt content. It can be seen that the void ratio of PAC is decreased with the increase in asphalt content. When asphalt content is 6%, all samples can satisfy the minimum void ratio of 18%, except the samples of “4% SBS + 10% CR” and “4% SBS”. More clearly, as shown in Table 3, the sample with the void ratio of “4% SBS + 20% CR” (6% asphalt content) has been taken as the design criteria to calculate asphalt content of each binder design. Besides, drain-down tests have been conducted to verify the mix proportions. Table 3 shows that drain-down test results of each mixed proportion design which required no more than 0.3% for the upper limit as specified in the Chinese protocol [32].

### 3.3. Engineering Performance Test 

**Figure 6:**
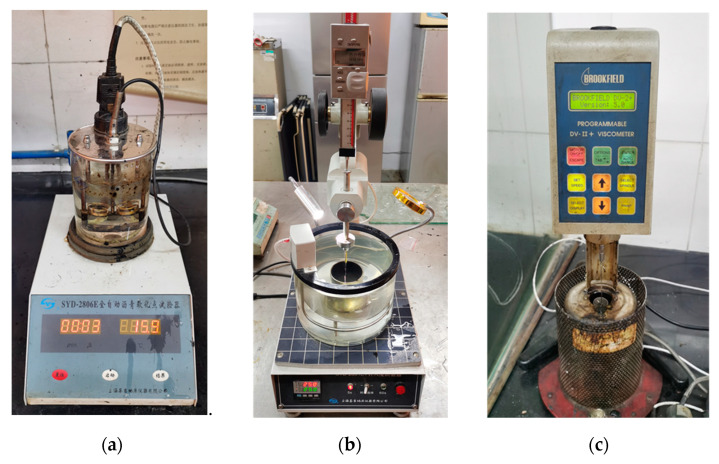
Test facilities for engineering test programs: (**a**) softening point test; (**b**) penetration test; (**c**) rotational viscosity test.

Three conventional engineering property tests, including softening point, penetration, and rotational viscosity tests, have been conducted in this study. Correspondingly, the protocols of ASTM D36 [34], ASTM D5 [35], and ASDM D4402 [36] have been followed to finish these test programs. Note that the unit of penetration and softening point are 0.1 mm and °C, respectively. Three temperatures (135 °C, 160 °C, 165 °C) have been adopted for the rotational viscosity test. All the tests have been conducted three times in parallel tests. Figure 6 shows the test facilities. 

### 3.4. MSCR Test 

**Figure 7:**
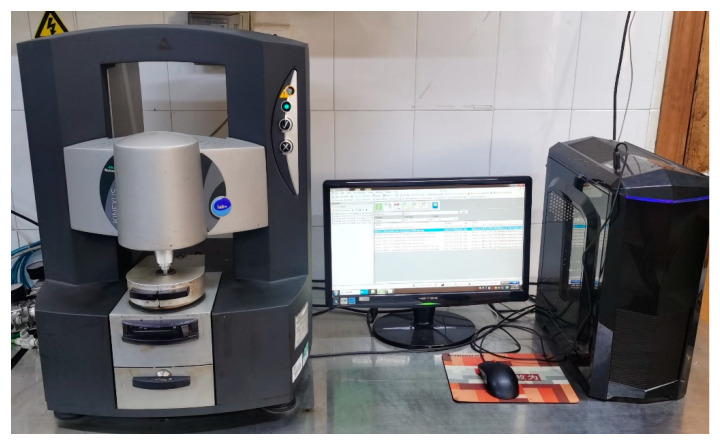
Test facility for MSCR and LAS test programs.

To investigate the rutting performance of the asphalt binder samples, the MSCR test, which is a creep and recovery test to assess the sample’s potential for permanent deformation, following AASHTO TP 70-10, has been conducted. Specifically, a Dynamic Shear Rheometer (DSR) has been adopted for the experimental test. For sample preparation, the diameter and thickness of the sample were 25 mm and 1 mm, respectively. A haversine shear load was applied to the sample for each loading cycle. The loading time was 1 s and the rest time was 9 s. There were a total of 10 cycles in each sample test and two stress levels, which were 0.1 KPa and 3.2 KPa, respectively. The indicators of non-recoverable creep compliance (*J_nr_*) and percentage recovery (*R*), which can be calculated in Equations (1) and (2), have been used to evaluate the rutting potential. Figure 7 shows the test equipment.

(1) $$R=\frac{\gamma_{p}-\;\gamma_{n}}{\gamma_{p}-\;\gamma_{0}}$$

(2) $$J_{nr}=\frac{\gamma_{n}-\;\gamma_{0}}{\tau }$$

Where *γ_p_* is the peak strain after one second creep duration, *γ*_0_ is the shear strain in the beginning of each loading cycle, *γ_n_* is the non-recoverable strain after nine seconds rest, and τ is the loading stress. 

### 3.5. Linear Amplitude Sweep (LAS) Test 

To evaluate the test samples’ fatigue resistance performance, an LAS test was conducted. Specifically, the LAS test is two-fold: the first step is to conduct the frequency sweep test for the evaluation of rheological characteristic of undamaged asphalt binder sample; the second step is to perform the linear amplitude strain sweep test. In detail, the frequency sweep test follows the AASHTO TP101. The strain amplitude is 0.1% and the frequency range is [0.2 Hz, 30 Hz]. A constant frequency of 10 Hz was adopted for the amplitude sweep test. Moreover, an 8 mm diameter parallel plate with 2 mm gap was applied on the DSR device. The load linearly increased from 0 to 30%. Note that all the testing samples needed to be conducted to PAV aging before the LAS test. Equation (3) presents the calculation of the number of cycles to failure (*N_f_*) at different strain levels. Figure 7 shows the test equipment.

(3) $$N_{f}=A{\left(\gamma_{max}\right)}^{-B}$$

Where *γ_max_* is the maximum strain. The calculations of *A* and *B* are outlined in detail elsewhere [37].

### 3.6. Freeze–Thaw Splitting Test

**Figure 8:**
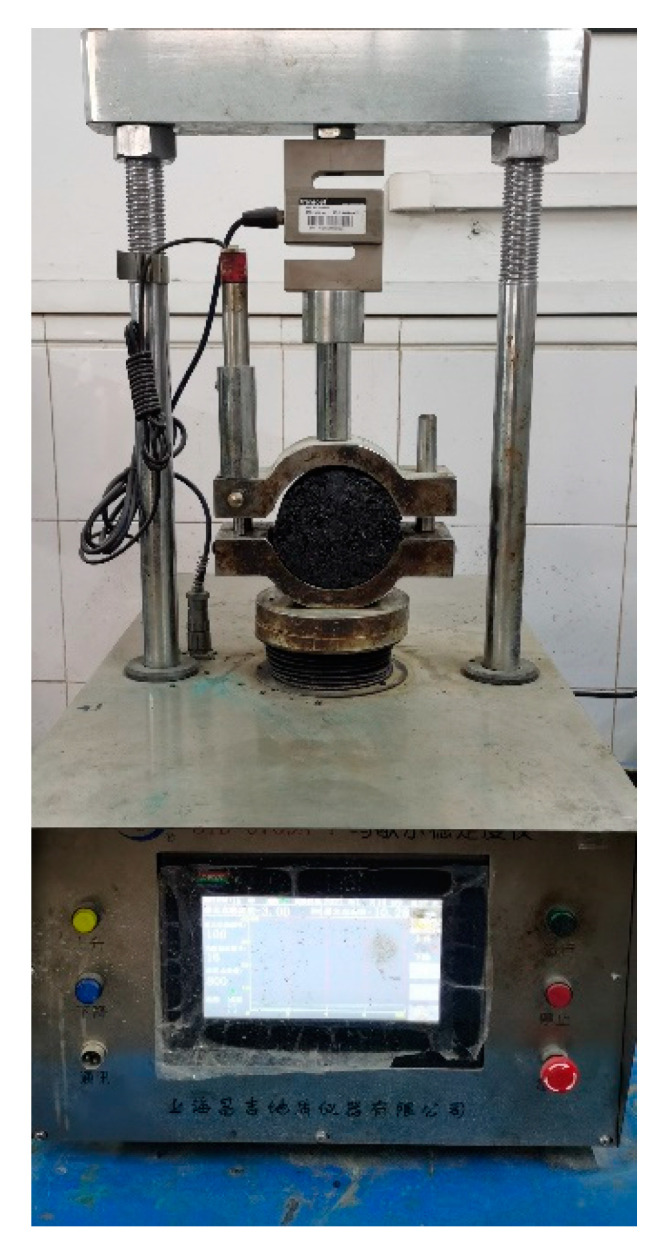
Test facility for freeze–thaw splitting test.

In order to investigate the moisture stability of SC-PAC, a freeze–thaw splitting test was conducted based on the Chinese protocol (E20-T0729-2011) [32]. Specifically, the well-prepared mixture samples needed to be kept in a vacuum with 97.3 KPa to 98.7 KPa pressure for 15 min. After that, the samples were kept in a water tank under normal pressure for another 30 min. The plastic bags needed to be prepared in advance before the submersion of the sample in the water. For each plastic bag, 10 mL of water and the Marshall sample were put together. Before putting the plastic bag into a refrigerator under a temperature of −18 °C for 16 h, there was a need to double check if there were holes around the plastic bag surface. The plastic bag was removed when the continuous freeze process was finished. Meanwhile, the mixture samples were put into hot water at 60 °C for 24 h. The splitting test was performed when the mixture samples were removed from the hot water. It needs to mention that the loading rate was 50 mm/min until the maximum load was reached. Figure 8 shows the test equipment. 

### 3.7. Hamburg Wheel-Tracking Test

**Figure 9:**
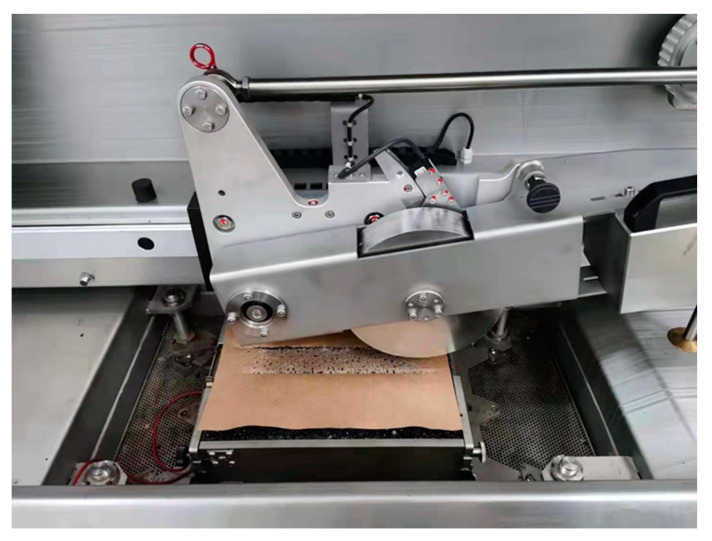
Test facility for the Hamburg wheel-tracking test.

To investigate the rutting resistance of SC-PAC, a Hamburg wheel-tracking test based on the Chinese specification [32] has been performed. The test device can be seen in Figure 9. The test samples were formulated in a square slab with a length, width, and height of 300 mm, 300 mm, and 50 mm, respectively. The weight of the rubber wheel was 78 kg. In this study, the test temperature was set as 45 °C, and the pressure applied on the sample surface was 0.7 MPa. During the Hamburg wheel-tracking test, the rubber wheel rolled back and forth on the sample surface for 1 h; meanwhile, the speed was launched at 161 mm/s. The rutting depth is utilized to evaluate different samples’ rutting resistance. Figure 9 shows the test equipment. 

### 3.8. Resilient Modulus Test

**Figure 10:**
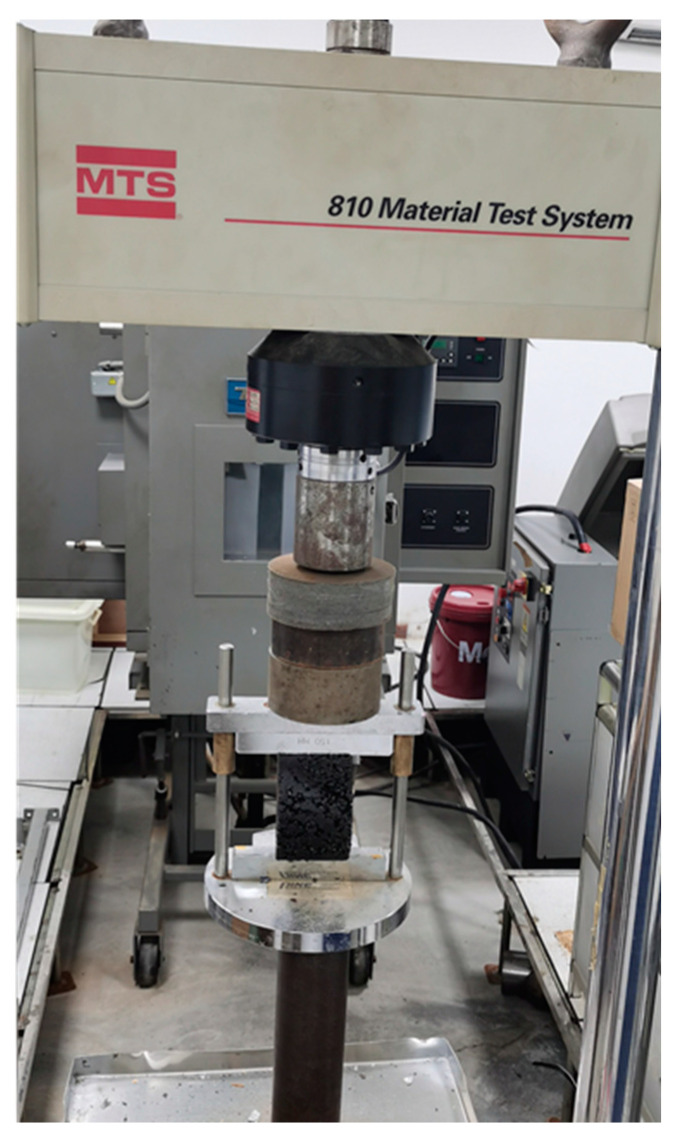
Test facility for resilient modulus test.

In order to investigate, evaluate, and compare the elastic property of the developed SC-PAC, the resilient modulus test based on the Chinese standard JTG E20-T0713-2011 [32] has been carried out with the uniaxial compression manner. In detail, the loading rate was set up to 2 mm/min, and environmental temperature was used to measure the compressive strength (*P*) for the first time (around 25 °C). Each loading was applied to the sample in seven incremental steps, progressing at 0.1 P, 0.2 P, 0.3 P, …, 0.7 P, respectively. Furthermore, with a time of 30 s for unloading, the resilient deformation of each loading stage was recorded in the test log file. The test equipment is displayed in Figure 10. After the resilient modulus test, the calculation of compressive strength and resilient deformation at 0.5 P can be shown in Equations (4) and (5) as follows:

(4) $$R_{c}=\frac{4P}{\pi d^{2}}$$

(5) $$E=\frac{q_{5\;\times h}}{\Delta L_{5}}$$

where *R_c_* is the compressive strength (MPa), *d* represents the diameter of the test sample, *P* denotes the peak loading (N), *h* represents the sample height (mm), *E* is the compressive resilient modulus (MPa), *q*_5_ denotes the compressive strength at the loading of 0.5 P, and ∆*L*_5_ is the resilient deformation (mm). 

### 3.9. Permeability Coefficient Test

The permeability is a primary indicator to assess the flow rate of water through the asphalt mixture. In this study, based on the Chinese protocol [32], the permeability coefficient test was conducted. Five types of samples were prepared and all samples have been tested three times. During calculation, the standard time was based on the period of the position change of water surface dropping from 100 mL to 500 mL. Specifically, if the seepage time is too long (i.e., over 3 min), we used the water volume at 3 min to calculate the permeability coefficient. The computation is shown in Equation (6) as follows:

(6) $$c_{w}=\;\frac{\left(\nu_{2}-\;\nu_{1}\right)\times 60}{\left(t_{2}-\;t_{1}\right)}$$

where *c_w_* is the permeability coefficient (mL/min), *ν*_1_ is the water volume in the first measurement (mL), *ν*_2_ is the water volume in the second measurement (mL), *t*_1_ refers to the time of the first measurement, and *t*_2_ indicates the time of the second measurement. 

## 4. Results Analysis and Discussion

### 4.1. Results of the Engineering Property Test 

**Figure 11:**
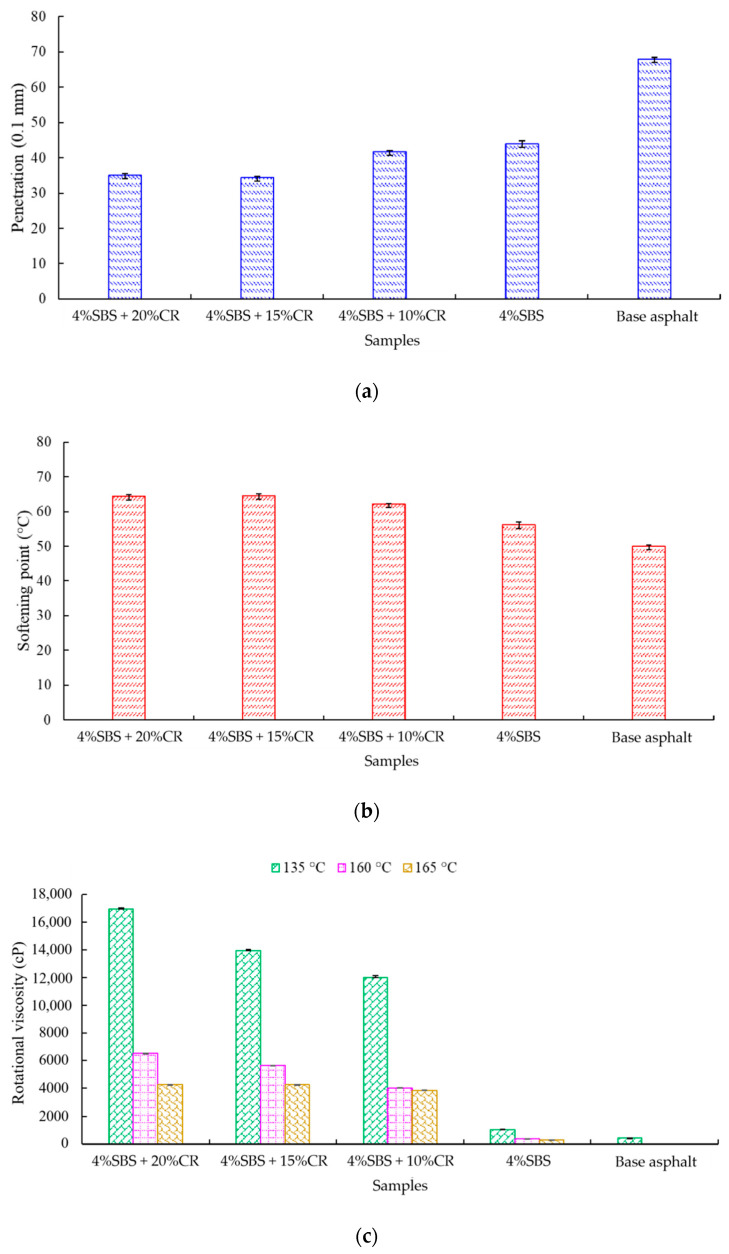
Results of engineering property tests: (**a**) penetration result; (**b**) softening point result; (**c**) rotational viscosity result.

As shown in Figure 11, three tests including penetration results, softening point results, and rotational viscosity results under three temperatures have been summarized. In Figure 11a, it is easy to conclude that with the addition of the CR, the penetration becomes lower compared to the virgin asphalt binder. The penetration of the virgin asphalt binder was 6.8 mm, while the penetration of the sample which had the lowest penetration (4% SBS + 20% CR; 3.5 mm) dropped 48.5%. This indicates the CR can make the asphalt binder harder compared to the normal mixture, indicating a good engineering performance. 

As for the result of softening point test, the minimum value (50 °C) was from the sample of the virgin asphalt binder, and the sample of the modified asphalt binder (4% SBS + 20% CR) had the maximum value of 64.4 °C, which was 28.8% higher that of the lowest value. The increasing trend of softening point with the increased ratio of CR also indicates good high-temperature properties of the modified asphalt binder. 

With respect to the result of the rotational viscosity test, under a temperature of 135 °C, the modified asphalt binder (4% SBS + 20% CR) had a super high viscosity, of 16,962.5 cP, while the virgin asphalt binder only had a value of 404.5 cP. It is easy to conclude that the CR contributed to the increase in the viscosity. Even under high temperature (165 °C), the rotational viscosity of the modified asphalt binder (4% SBS + 20% CR) was still high viscosity; 4290 cP.

### 4.2. Results of the Multiple Stress Creep Recovery (MSCR) Test

**Figure 12:**
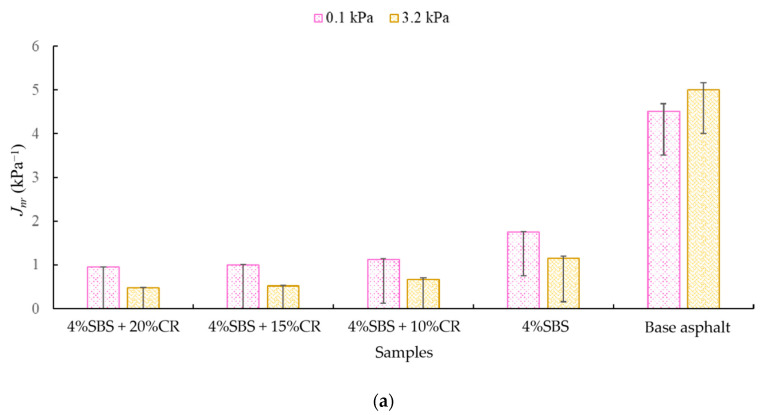

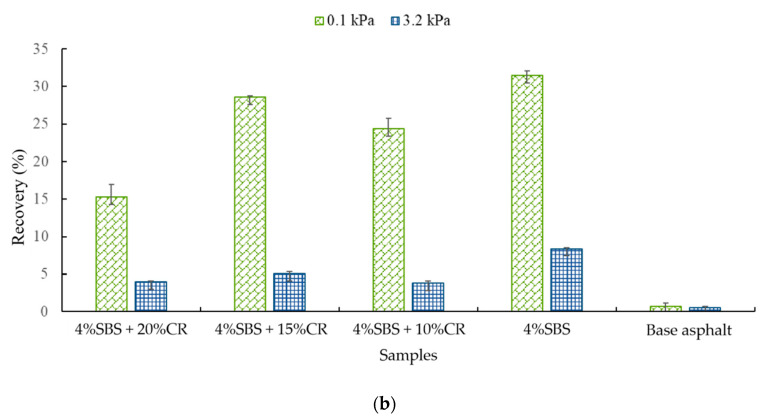
MSCR results: (**a**) *J_nr_* (kPa^−1^) and (**b**) Recovery (%).

Regarding the results of the MSCR test, two indicators (*J_nr_* and *R*) mentioned in Section 3 have been adopted. Zhang et al. [38] have pointed out that a lower value of *J_nr_* suggests a higher rutting resistance. Meanwhile, Hossain et al. [39] have reported that *R* is a measurement of identification and quantification of polymers working in the asphalt binder. As shown in Figure 12a, under the stress level of 0.1 kPa, the sample with 4% SBS + 20% CR had the minimum *J_nr_* value, which was 0.952, 78.9% lower compared to the *J_nr_* value of the virgin binder. This reveals a much better rutting resistance of the modifier asphalt binder compared to the original. Under the stress level of 3.2 kPa, our modified asphalt binder still had the minimum *J_nr_* value (0.483), 90.3% lower compared to the virgin asphalt binder, indicating a better rutting resistance under the high stress level. Note that with the addition of CR, the *J_nr_* value continually decreased. At the opposite angle, the rutting resistance performance of our modified asphalt binder still shows promise. 

With respect to the percentage recovery, *R*, Figure 12b clearly presents the content change of the polymer in the asphalt binder. With the low stress level (0.1 kPa), the addition of SBS increased the recovery, while when the CR was included, the recovery decreased. A similar scenario occurred under the high stress level (3.2 kPa). To sum up, the CR can sacrifice the recovery, but the ability to resist permanent damage is still considerably higher than the virgin asphalt binder. The reason behind this deserves further exploration in our future theoretical study with chemical approaches. 

### 4.3. Results of the Linear Amplitude Sweep (LAS) Test 

**Figure 13:**
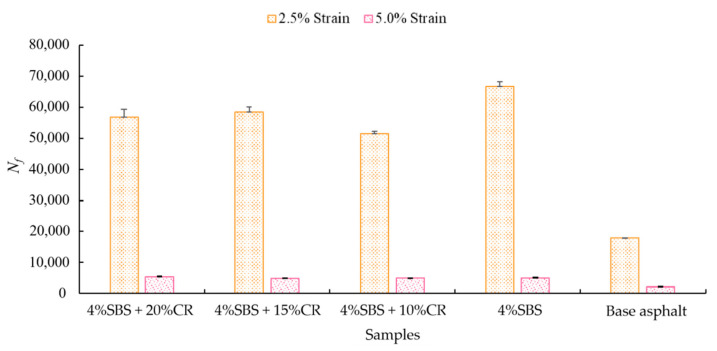
Fatigue performance of different samples.

As discussed in Sabouri’s study [40], a higher value of *N_f_* suggests a better fatigue performance. Figure 13 depicts the fatigue performance of the different tested samples under two different strain levels (2.5% and 5.0%). Specifically, under the 2.5% strain condition, with the addition of SBS, the fatigue life improved 271% first. Then, the sample which included 10% CR made the fatigue life drop by 22.6%. An even greater content of CR can contribute to the extended fatigue life; although it is lower than the asphalt binder with SBS only. As for the test condition under 5% strain, the SBS can induce a 126% increase in fatigue life, but it is 6.4% lower compared to the modified asphalt binder with 4% SBS + 20% CR. Interestingly, the modified asphalt binder with 4% SBS + 20% CR showed limited fatigue performance. It was 11.5% lower compared to the modified asphalt binder with 4% SBS + 20% CR. Overall, our modified asphalt binder (4% SBS + 20% CR) showed the best fatigue performance. 

### 4.4. Results of the Freeze–Thaw Splitting Test 

**Figure 14:**
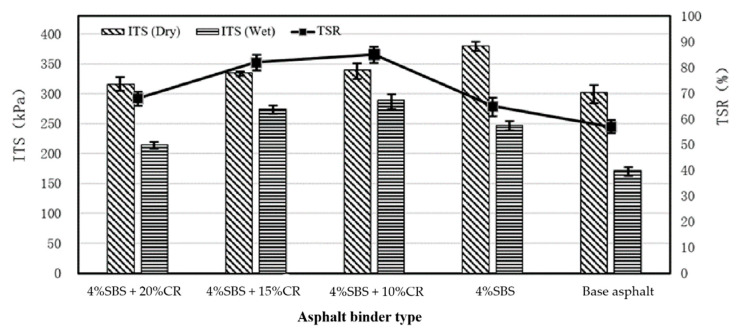
Results of freeze–thaw splitting test (indirect tensile strength (ITS) and tensile strength ratio (TSR)).

Typically, the PAC has a macro porous structure compared to the conventional asphalt mixture, indicating a weak performance regarding the resistance of moisture damage and cracking. Figure 14 shows the results of freeze–thaw splitting tests using the indirect tensile strength (ITS) and tensile strength ratio (TSR) to evaluate the moisture stability and cracking resistance of different kinds of PAC samples. 

As shown in Figure 14, compared to the PAC sample using virgin asphalt binder, the SBS modified PAC sample can significantly improve the TSR values, indicating better moisture stability and cracking resistance on PAC designs. When using 10% CR and 4% SBS on PAC, it can be found that the TSR increases by 34.6%, and the ITS (dry) slightly increases by 13.8%. Unfortunately, with more SBS modifiers and CRs, the moisture stability becomes worse, associated with the dropped values on either the ITS indicator or TSR indicator. With 4% SBS and 10% CR, the PAC design had the optimum performance on the moisture stability and cracking resistance. However, to meet the requirement that the minimum TSR value should be 80%, further optimizations and investigations will be conducted. 

### 4.5. Results of the Hamburg Wheel-Tracking Test

**Figure 15:**
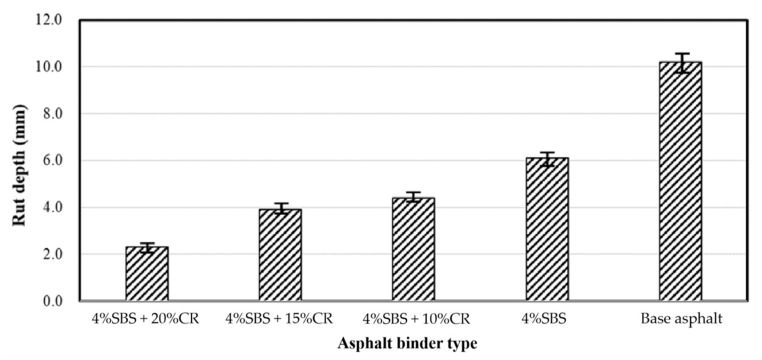
Wheel-tracking test results of different PACs.

As for the PAC samples, there was less contact between aggregate particles compared to the mixture structure of dense asphalt concrete and stone matrix asphalt (SMA); therefore, the rutting resistance performance under high-temperature service needs to be explored. Figure 15 presents the results of the Hamburg wheel-tracking test with the different aforementioned PAC designs. Apparently, the PAC with the virgin asphalt binder had the maximum wheel-tracking depth, which was 11 mm. Meanwhile, the PAC with 4% SBS and 20% CR had the minimum wheel-tracking depth, which was 2.2 mm, 400% lower compared to the PAC with the virgin asphalt binder. Moreover, with the addition of the CR, the wheel-tracking depth decreased, indicating that CR can be beneficial to the high-temperature performance of PAC. The reasons that the CR and SBS modifiers can help improve the high-temperature performance are that the CR itself has better recovery ability, and the SBS modifier can contribute to the generation of the three-dimensional network structure to improve the viscoelasticity property of the virgin asphalt binder. 

### 4.6. Results of the Resilient Modulus Test 

**Figure 16:**
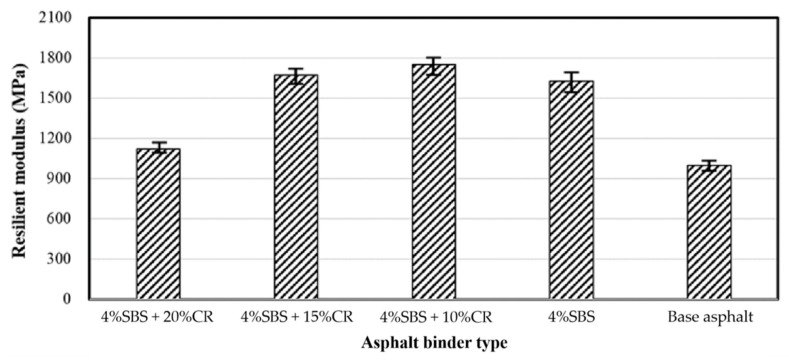
Resilient test results of different PACs.

The resilient test results of different PACs have been plotted in Figure 16. The PAC containing 4% SBS and 10% CR had the maximum resilient modulus value, which was 1740 MPa. The PAC with the virgin asphalt binder demonstrated the lowest resilient modulus, which was 1010 MPa. With the addition of 4% SBS modifier, the PAC’s resilient modulus reached 1580 MPa, 56.4% higher compared to the PAC with virgin asphalt binder, but 10% lower regarding the PAC with 4% SBS modifier and 10% CR. Interestingly, more CR would induce a decrease in the resilient modulus of the PAC. The PAC with 4% SBS modifier and 15% CR obtained the resilient modulus value of 1680 MPa, 4.7% lower compared to the PAC with 4% SBS modifier and 10% CR. The reason can be explained from two points: (i) under a certain content range of the CR, the addition of the CR can increase the elasticity of the asphalt mixture, therefore lowering the stiffness of the PAC; (ii) when the content of the CR exceeds its limit, it will behave more like a fine aggregate. In other words, the void content in the PAC is reduced and then the overall modulus of the asphalt mixture drops, showing that a high CR content brings a low resilient modulus. 

### 4.7. Results of the Permeability Coefficient Test 

**Figure 17:**
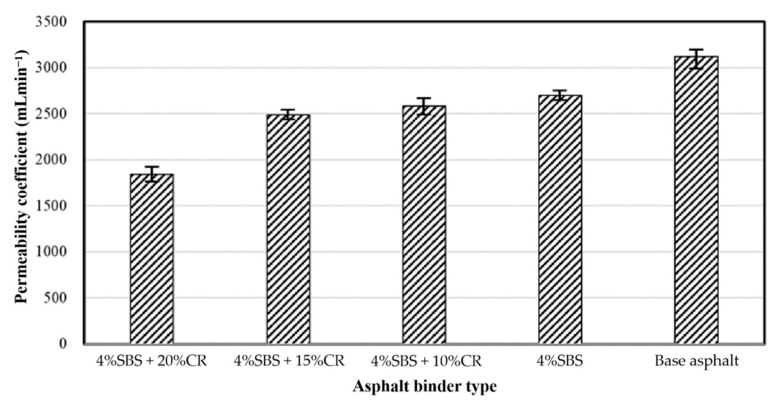
Permeability test results of different PACs.

Figure 17 presents the permeability test results of different PACs. It is clear to see that with more SBS and CR in the PAC, the permeability decreases. The PAC manufactured with the virgin asphalt binder showed the maximum permeability coefficient value, which was 3126 mL·min^−1^. With the addition of CR, the PACs’ permeability coefficient value decreased. When there was 20% CR in the PAC, there was the minimum permeability coefficient of PAC; 1835 mL·min^−1^. It is easy to understand and explain this scenario because the CR particle is more like a small aggregate that can clog the void path of the PAC. The proportion of connected voids decreases, and then the permeability coefficient decreases. In addition, a further discussion on the impact of void ration on permeability coefficient will be conducted in the future.

## 5. Conclusions 

This study has investigated the asphalt binder properties and the asphalt mixture properties of PAC with 4% SBS and three different dosages of CR. Thorough experimental programs evaluating the high temperature performance, fatigue performance, crack resistance performance, and water resistance performance have been performed. The observations, results, and findings can be concluded as follows.

1. Given the same void ratio, the optimum asphalt content of PAC gradually increases along with the increased content of CR. However, the impact of the SBS modifier and 10% CR on the optimum asphalt content is not significant;
2. The CR and SBS modifier can improve the high temperature performance, rutting performance and fatigue performance regarding the results of engineering property test, MSCR test and LAS test. A 15% content of CR is preferable;
3. The addition of CR and SBS modifier can improve the water stability and crack resistance of PAC, but the content of CR should be appropriate and preferably controlled between 10% and 15%;
4. Adding CR and the SBS modifier can significantly improve the rutting resistance and resilience modulus of PAC, but CR in a high content (generally over 15%) can significantly reduce the resilience modulus of PAC. Therefore, the content of CR should be preferably controlled below 15% when producing PAC;
5. Given the same void ratio, the permeability coefficient of the CR/SBS composite modified PAC is lower than that of conventional matrix PAC. Therefore, a proper way to ensure the water permeability of CR/SBS composite modified PAC is to increase its design void ratio.

Finally, the material design of 4% SBS + 10% CR has been successfully applied in a field project in Shaoguan, Guangdong Province. A more in-depth study about the relationship between field performance and experimental data in the lab will be conducted in future.

## Figures and Tables

**Table 1:** Technical indicators of base asphalt.

Test Item	Test Result	Test Method
Penetration (0.1 mm)	68	T0604
Softening Point (°C)	50	T0606
Ductility (25 °C) (cm)	125	T0605
Density (25 °C) (g/cm^3^)	1.048	T0603

**Table 2:** Gradations of crumb rubber (CR).

Sieve Size (mm)	Passing Rate (%)
1.18	100
0.6	98.2
0.3	82
0.15	7
0.075	0

**Table 3:** Optimum asphalt content and drain-down rate.

Binder Type	4% SBS + 20% CR	4% SBS + 15% CR	4% SBS + 10% CR	4% SBS	Base Asphalt
Optimum Asphalt Content	6%	5.8%	5.4%	5.3%%	5.4%
Drain-Down Rate	0.11%	0.13%	0.16%	0.20%	0.46%

## Data Availability

The data supporting the findings of this research is available within the article.
